# Supplementary material for: Hot spot prediction in protein-protein interactions by an ensemble system
Source: BMC Syst Biol. 2018 Dec 31;12(Suppl 9):132. doi: 10.1186/s12918-018-0665-8 (PMC6311905; doi:10.1186/s12918-018-0665-8)
Supplement: Supplementary file 2 — All relevant MATLAB code. A simple Matlab implement of our predictor. (ZIP 19,742 kb) [file 12918_2018_665_MOESM2_ESM.zip › Additional file 2/Read ASA/ASA Property/BID/1CDLA.txt]

 NetSurfP - Protein Surface Accessibility and Secondary Structure Predictions

  

|  |  |  |
| --- | --- | --- |
|  |  | NetSurfP - Protein Surface Accessibility and Secondary Structure Predictions  Technical University of Denmark |

  


---

```
# For publication of results, please cite:
# A generic method for assignment of reliability scores applied to solvent accessibility predictions.
# Bent Petersen, Thomas Nordahl Petersen, Pernille Andersen, Morten Nielsen and Claus Lundegaard
# BMC Structural Biology 2009, 9:51 doi:10.1186/1472-6807-9-51
#
# Column 1: Class assignment - B for buried or E for Exposed - Threshold: 25% exposure, but not based on RSA
# Column 2: Amino acid
# Column 3: Sequence name
# Column 4: Amino acid number
# Column 5: Relative Surface Accessibility - RSA
# Column 6: Absolute Surface Accessibility
# Column 7: Z-fit score for RSA prediction
# Column 8: Probability for Alpha-Helix
# Column 9: Probability for Beta-strand
# Column 10: Probability for Coil
E A  1CDLA                  1    0.631  69.569  -1.284   0.003   0.003   0.994
E D  1CDLA                  2    0.744 107.268   0.827   0.053   0.005   0.942
E Q  1CDLA                  3    0.692 123.591   1.531   0.053   0.005   0.942
B L  1CDLA                  4    0.096  17.596   0.566   0.016   0.005   0.979
E T  1CDLA                  5    0.442  61.236   2.091   0.016   0.005   0.979
E E  1CDLA                  6    0.695 121.382   1.788   0.923   0.002   0.076
E E  1CDLA                  7    0.699 122.098   1.489   0.923   0.002   0.076
B Q  1CDLA                  8    0.161  28.826   0.868   0.923   0.002   0.076
B I  1CDLA                  9    0.136  25.123   1.199   0.970   0.001   0.030
E A  1CDLA                 10    0.578  63.652   1.705   0.970   0.001   0.030
E E  1CDLA                 11    0.366  63.870   2.183   0.970   0.001   0.030
B F  1CDLA                 12    0.014   2.750   1.736   0.988   0.000   0.012
E K  1CDLA                 13    0.301  61.998   2.115   0.988   0.000   0.012
E E  1CDLA                 14    0.480  83.769   1.869   0.988   0.000   0.012
B A  1CDLA                 15    0.054   5.940   1.417   0.970   0.001   0.030
B F  1CDLA                 16    0.021   4.175   1.271   0.970   0.001   0.030
E S  1CDLA                 17    0.494  57.909   2.084   0.923   0.002   0.076
E L  1CDLA                 18    0.318  58.299   1.708   0.879   0.010   0.111
B F  1CDLA                 19    0.042   8.409   1.025   0.717   0.014   0.269
B D  1CDLA                 20    0.088  12.738  -0.021   0.430   0.016   0.555
E K  1CDLA                 21    0.637 130.990   0.550   0.246   0.004   0.750
E D  1CDLA                 22    0.556  80.120   0.107   0.053   0.005   0.942
E G  1CDLA                 23    0.577  45.434  -1.127   0.016   0.005   0.979
E D  1CDLA                 24    0.602  86.734  -0.739   0.003   0.003   0.994
E G  1CDLA                 25    0.237  18.620  -1.102   0.004   0.085   0.910
E T  1CDLA                 26    0.393  54.481   1.721   0.005   0.336   0.660
B I  1CDLA                 27    0.031   5.680   0.653   0.022   0.411   0.567
E T  1CDLA                 28    0.257  35.701   1.440   0.018   0.088   0.893
B T  1CDLA                 29    0.177  24.481   0.996   0.923   0.002   0.076
E K  1CDLA                 30    0.634 130.332   1.460   0.970   0.001   0.030
B E  1CDLA                 31    0.101  17.575   0.694   0.970   0.001   0.030
B L  1CDLA                 32    0.014   2.508   1.602   0.970   0.001   0.030
B G  1CDLA                 33    0.181  14.276   1.234   0.970   0.001   0.030
E T  1CDLA                 34    0.428  59.294   1.743   0.970   0.001   0.030
B V  1CDLA                 35    0.030   4.580   0.351   0.970   0.001   0.030
B M  1CDLA                 36    0.028   5.523   0.726   0.970   0.001   0.030
E R  1CDLA                 37    0.395  90.409   1.084   0.970   0.001   0.030
E S  1CDLA                 38    0.402  47.091   0.384   0.923   0.002   0.076
B L  1CDLA                 39    0.083  15.234   0.421   0.600   0.003   0.397
E G  1CDLA                 40    0.389  30.638  -1.081   0.089   0.000   0.910
B Q  1CDLA                 41    0.273  48.686   0.292   0.053   0.005   0.942
E N  1CDLA                 42    0.579  84.736  -0.389   0.016   0.005   0.979
B P  1CDLA                 43    0.127  18.021   0.081   0.003   0.003   0.994
E T  1CDLA                 44    0.445  61.694   0.660   0.016   0.005   0.979
E E  1CDLA                 45    0.511  89.324   0.338   0.923   0.002   0.076
E A  1CDLA                 46    0.597  65.811   0.986   0.970   0.001   0.030
B E  1CDLA                 47    0.195  34.084   1.005   0.970   0.001   0.030
B L  1CDLA                 48    0.015   2.820   1.336   0.988   0.000   0.012
E Q  1CDLA                 49    0.373  66.636   2.057   0.988   0.000   0.012
E D  1CDLA                 50    0.470  67.741   2.174   0.988   0.000   0.012
B M  1CDLA                 51    0.035   7.004   0.650   0.970   0.001   0.030
B I  1CDLA                 52    0.043   7.973   0.031   0.970   0.001   0.030
E N  1CDLA                 53    0.566  82.892   1.313   0.938   0.007   0.055
E E  1CDLA                 54    0.450  78.580   0.784   0.879   0.010   0.111
B V  1CDLA                 55    0.052   7.962   0.347   0.622   0.015   0.363
E D  1CDLA                 56    0.249  35.895   0.096   0.176   0.004   0.820
E A  1CDLA                 57    0.583  64.269  -0.738   0.109   0.005   0.886
E D  1CDLA                 58    0.651  93.867  -0.789   0.016   0.005   0.979
E G  1CDLA                 59    0.633  49.841  -2.126   0.016   0.005   0.979
E N  1CDLA                 60    0.686 100.504  -1.756   0.003   0.003   0.994
E G  1CDLA                 61    0.374  29.402  -0.536   0.005   0.045   0.951
E T  1CDLA                 62    0.413  57.283   0.666   0.005   0.262   0.733
B I  1CDLA                 63    0.032   5.883   0.561   0.022   0.411   0.567
E D  1CDLA                 64    0.326  46.962   1.437   0.005   0.262   0.733
B F  1CDLA                 65    0.055  11.059   1.093   0.879   0.010   0.111
E P  1CDLA                 66    0.512  72.710   0.866   0.970   0.001   0.030
B E  1CDLA                 67    0.169  29.559   0.656   0.970   0.001   0.030
B F  1CDLA                 68    0.019   3.813   0.994   0.970   0.001   0.030
B L  1CDLA                 69    0.072  13.110   0.909   0.975   0.003   0.022
E T  1CDLA                 70    0.367  50.958   1.297   0.970   0.001   0.030
B M  1CDLA                 71    0.045   8.904   0.300   0.970   0.001   0.030
B M  1CDLA                 72    0.028   5.603   0.359   0.970   0.001   0.030
B A  1CDLA                 73    0.227  24.971   0.274   0.970   0.001   0.030
E R  1CDLA                 74    0.452 103.439   0.228   0.923   0.002   0.076
B K  1CDLA                 75    0.195  40.153  -0.229   0.858   0.002   0.139
B M  1CDLA                 76    0.287  57.429  -0.478   0.600   0.003   0.397
E K  1CDLA                 77    0.697 143.373  -0.523   0.321   0.003   0.675
E D  1CDLA                 78    0.585  84.299  -1.384   0.109   0.005   0.886
E T  1CDLA                 79    0.549  76.146  -0.801   0.053   0.005   0.942
E D  1CDLA                 80    0.539  77.641  -0.554   0.016   0.005   0.979
E S  1CDLA                 81    0.404  47.407   0.767   0.502   0.002   0.495
E E  1CDLA                 82    0.392  68.535   0.344   0.970   0.001   0.030
E E  1CDLA                 83    0.550  96.085   1.300   0.970   0.001   0.030
B E  1CDLA                 84    0.207  36.215   1.850   0.988   0.000   0.012
B I  1CDLA                 85    0.015   2.830   1.751   0.988   0.000   0.012
E R  1CDLA                 86    0.275  62.906   1.945   0.988   0.000   0.012
E E  1CDLA                 87    0.440  76.938   1.587   0.970   0.001   0.030
B A  1CDLA                 88    0.027   3.030   0.620   0.975   0.003   0.022
B F  1CDLA                 89    0.023   4.536   1.010   0.938   0.007   0.055
E R  1CDLA                 90    0.433  99.157   1.910   0.802   0.014   0.185
B V  1CDLA                 91    0.228  35.028   0.936   0.660   0.049   0.291
B F  1CDLA                 92    0.043   8.690   0.871   0.307   0.165   0.527
B D  1CDLA                 93    0.089  12.767  -0.557   0.052   0.084   0.864
E K  1CDLA                 94    0.562 115.583   0.584   0.058   0.017   0.925
E D  1CDLA                 95    0.642  92.498  -0.158   0.016   0.005   0.979
E G  1CDLA                 96    0.611  48.101  -1.284   0.003   0.003   0.994
E N  1CDLA                 97    0.608  89.084  -0.835   0.003   0.003   0.994
B G  1CDLA                 98    0.242  19.077  -1.383   0.005   0.045   0.951
E Y  1CDLA                 99    0.366  78.150   1.376   0.005   0.336   0.660
B I  1CDLA                100    0.026   4.902   0.743   0.005   0.336   0.660
E S  1CDLA                101    0.252  29.534   1.197   0.018   0.088   0.893
B A  1CDLA                102    0.199  21.974   0.806   0.970   0.001   0.030
E A  1CDLA                103    0.646  71.211   1.206   0.970   0.001   0.030
B E  1CDLA                104    0.162  28.232   1.092   0.970   0.001   0.030
B L  1CDLA                105    0.012   2.216   1.638   0.970   0.001   0.030
B R  1CDLA                106    0.216  49.418   1.887   0.975   0.003   0.022
E H  1CDLA                107    0.467  84.893   2.143   0.970   0.001   0.030
B V  1CDLA                108    0.038   5.887   0.329   0.970   0.001   0.030
B M  1CDLA                109    0.026   5.143   0.574   0.970   0.001   0.030
E T  1CDLA                110    0.456  63.219   1.549   0.970   0.001   0.030
E N  1CDLA                111    0.529  77.431   1.339   0.923   0.002   0.076
B L  1CDLA                112    0.153  28.033   0.144   0.600   0.003   0.397
E G  1CDLA                113    0.339  26.695  -0.980   0.089   0.000   0.910
E E  1CDLA                114    0.493  86.110   0.273   0.176   0.004   0.820
E K  1CDLA                115    0.647 133.088   0.641   0.176   0.004   0.820
B L  1CDLA                116    0.071  12.927   0.657   0.018   0.019   0.964
E T  1CDLA                117    0.382  52.928   1.081   0.016   0.005   0.979
E D  1CDLA                118    0.513  73.967   0.909   0.970   0.001   0.030
E E  1CDLA                119    0.617 107.790   1.704   0.970   0.001   0.030
B E  1CDLA                120    0.175  30.660   1.791   0.970   0.001   0.030
B V  1CDLA                121    0.016   2.490   1.656   0.988   0.000   0.012
E D  1CDLA                122    0.366  52.769   2.279   0.988   0.000   0.012
E E  1CDLA                123    0.438  76.554   2.325   0.988   0.000   0.012
B M  1CDLA                124    0.030   6.023   1.095   0.970   0.001   0.030
B I  1CDLA                125    0.024   4.477   1.275   0.975   0.003   0.022
E R  1CDLA                126    0.510 116.767   2.070   0.938   0.007   0.055
E E  1CDLA                127    0.395  69.076   1.575   0.879   0.010   0.111
B A  1CDLA                128    0.024   2.634   1.064   0.522   0.016   0.462
B D  1CDLA                129    0.173  24.900   0.196   0.109   0.005   0.886
E I  1CDLA                130    0.567 104.951  -0.083   0.053   0.005   0.942
E D  1CDLA                131    0.492  70.825  -0.370   0.016   0.005   0.979
E G  1CDLA                132    0.649  51.068  -1.185   0.003   0.003   0.994
E D  1CDLA                133    0.658  94.875  -0.855   0.003   0.003   0.994
E G  1CDLA                134    0.237  18.644  -0.786   0.005   0.045   0.951
E Q  1CDLA                135    0.414  73.976   1.623   0.005   0.336   0.660
B V  1CDLA                136    0.034   5.241   0.724   0.004   0.514   0.481
E N  1CDLA                137    0.324  47.434   1.631   0.021   0.279   0.699
B Y  1CDLA                138    0.122  25.986   1.066   0.970   0.001   0.030
E E  1CDLA                139    0.584 102.060   1.455   0.970   0.001   0.030
E E  1CDLA                140    0.314  54.891   1.423   0.970   0.001   0.030
B F  1CDLA                141    0.035   7.004   0.129   0.988   0.000   0.012
B V  1CDLA                142    0.173  26.652   1.826   0.975   0.003   0.022
E Q  1CDLA                143    0.520  92.801   1.485   0.938   0.007   0.055
B M  1CDLA                144    0.167  33.417   1.316   0.938   0.007   0.055
B M  1CDLA                145    0.093  18.509  -1.242   0.802   0.014   0.185
E T  1CDLA                146    0.455  63.053   0.217   0.522   0.016   0.462
E A  1CDLA                147    0.786  86.584  -2.253   0.003   0.003   0.994

Explain the output.  Go back.
```

---
